# Innovation in health service delivery: integrating community health assistants into the health system at district level in Zambia

**DOI:** 10.1186/s12913-015-0696-4

**Published:** 2015-01-28

**Authors:** Joseph Mumba Zulu, Anna-Karin Hurtig, John Kinsman, Charles Michelo

**Affiliations:** Department of Public Health, School of Medicine, University of Zambia, P.O. Box 50110, Lusaka, Zambia; Umeå International School of Public Health (UISPH), Umeå University, Umeå, SE 90185 Sweden

**Keywords:** Integration, Health innovations, Community-based health workers, Health system

## Abstract

**Background:**

To address the huge human resources for health gap in Zambia, the Ministry of Health launched the National Community Health Assistant Strategy in 2010. The strategy aims to integrate community-based health workers into the health system by creating a new group of workers, called community health assistants (CHAs). However, literature suggests that the integration process of national community-based health worker programmes into health systems has not been optimal. Conceptually informed by the diffusion of innovations theory, this paper qualitatively aimed to explore the factors that shaped the acceptability and adoption of CHAs into the health system at district level in Zambia during the pilot phase.

**Methods:**

Data gathered through review of documents, 6 focus group discussions with community leaders, and 12 key informant interviews with CHA trainers, supervisors and members of the District Health Management Team were analysed using thematic analysis.

**Results:**

The perceived relative advantage of CHAs over existing community-based health workers in terms of their quality of training and scope of responsibilities, and the perceived compatibility of CHAs with existing groups of health workers and community healthcare expectations positively facilitated the integration process. However, limited integration of CHAs in the district health governance system hindered effective programme trialability, simplicity and observability at district level. Specific challenges at this level included a limited information flow and sense of programme ownership, and insufficient documentation of outcomes. The district also had difficulties in responding to emergent challenges such as delayed or non-payment of CHA incentives, as well as inadequate supervision and involvement of CHAs in the health posts where they are supposed to be working. Furthermore, failure of the health system to secure regular drug supplies affected health service delivery and acceptability of CHA services at community level.

**Conclusion:**

The study has demonstrated that implementation of policy guidelines for integrating community-based health workers in the health system may not automatically guarantee successful integration at the local or district level, at least at the start of the process. The study reiterates the need for fully integrating such innovations into the district health governance system if they are to be effective.

## Background

Human, material and financial resource scarcity, coupled with a high disease burden, have necessitated the development and adoption of several innovations aimed at improving health outcomes into the health systems of low and middle income countries [1]. Innovations in health systems refer to new ideas, initiatives, strategies, practices, medicines, diagnostics, and health technologies which are perceived as fresh by the adopting individual, institution or unit [1,2]. While some innovations are in hardware form, for example long-lasting insecticidal nets and antiretroviral treatment [3], others such as new human resources for health approaches can be seen as a critical part of the health system’s software [4].

The last decade has recorded an increase in human resources for health innovations. These include extending the role of some professional staff to undertake extra duties, and involving the private sector in the training of health workers [5]. Development and implementation of institutionalised or national community-based health worker programmes is another form of innovation. Unlike other community-based health worker programmes, such programmes have been formed and operated by the government; have training, supervision and incentive structures that are standardised and well-defined by the government; and have been scaled-up nationally [6]. Pakistan, India and Ethiopia are some of the countries that have formalised community-based health workers’ services. In Pakistan, this innovative approach is called the Lady Health Worker programme, while along similar lines, India has the Accredited Social Health Activist programme [7]. In Ethiopia, the institutionalised programme is known as the Health Extension Worker programme [8].

Compared to other community-based health workers, the institutionalised group has longer and standardised training, they perform more tasks, and they receive better supervision from professional health workers. As a result, institutionalised approaches have facilitated good health outcomes at community level. For example, the Lady Health Worker programme which delivers a package of integrated maternal, child health and family planning services door-to-door, has recorded increased utilisation of its services in Pakistan [9]. Similarly, the Health Extension Worker programme has helped reduce geographical barriers to care, and subsequently increased the percentage of births with skilled attendants, women receiving antenatal care, and fully immunized infants in Ethiopia [10].

Socio-cultural issues, availability of drugs, monetary support and individual behaviour of workers are some of the issues that have shaped the integration process .i.e. the extent, pattern and level of adoption of these innovations into the health system [4,9,11]. The quality and type of supervision was shown to be important in facilitating the integration of the Lady Health Worker programme in the health system in Pakistan [8]. Studies on the India’s Accredited Social Health Activists have shown that a clear definition of their responsibilities played a key role in shaping acceptability of their tasks by other stakeholders into the health system [7]. As for the Ethiopian Health Extension Workers, their integration process in health system was shaped by among other issues management capacity by supervisors, pay consistency and adequacy of the orientation process to communities regarding their role [12,13]. Integration of community-based health worker programmes into the health systems has also been influenced by the level of trust, appreciation and support by community and family members as well as the attitudes of professional health workers [14] .

In 2010, the Ministry of Health in Zambia also developed an innovative strategy to help address the critical shortage of human resources for health in the country. This strategy is intended to address the human resources for health gap by creating a new group of community-based health workers called community heath assistants (CHAs), who will be institutionalised within the health system depending on the results of the pilot phase [15]. Currently, Zambia has about half the health workforce that it needs, with fewer than 646 doctors and 6,096 nurses serving a national population of 14 million people. Vacancies among nursing cadres stand at 55%, with 63% and 64% of clinical officer and doctor posts unfilled respectively [16]. As a result, about 23,500 voluntary community-based health workers have been helping in providing primary health care [15].

This human resources for health innovation falls under the National Community Health Assistant strategy. Compared to other community-based health workers, whose training is short and not standardised, CHAs undergo a one-year standardised training programme, they are registered with a regulatory body, perform much broader tasks, and will be put on the government payroll. CHAs work below nurses and they deliver health services through a task-shifting approach (i.e. from nurse to CHA). The CHAs are supposed to spend 80% (four days in a week) of their work time in the community and 20% (one day in a week) at the health post [15,17].

Implementation of the strategy started in June 2011 with a pilot phase which ended in 2013. In the first phase, 307 CHAs were trained and deployed simultaneously in the health posts in seven provinces in August 2012 [15]. On average, two CHAs were deployed at each health post. At the health post CHAs perform several activities which include screening patients (taking vital signs), treating minor illnesses such as malaria, diarrhoea, respiratory tract infections and burns/sores and assisting with delivering children [15]. In the community, by contrast, CHAs conduct health promotion activities on the use of treated mosquito nets, how to maintain good sanitation standards, sensitisation of the community on how to prevent diarrhoea through boiling water for drinking and applying chlorine, as well as testing for and treating minor illnesses. CHAs also develop registers on the total number of people and common diseases in the community which are used for guiding the Ministry of Health in planning community health services [15].

Although there has been an increase in the number of countries integrating community-based health workers in the health systems, our systematic review on the integration process of national community-based health worker programmes into health systems in low and middle incomes showed that the integration has not been optimal [6]. Meanwhile, there is limited knowledge on the factors that shape the acceptability and adoption of such innovations at district level health system in low and middle income countries. Recent studies on institutionalised community-based health workers have focused more on their role in improving disease-specific outcomes [5,18,19], and management of institutionalised programs [7]. In Zambia, current studies on the CHA programme have focused on recruitment processes [20]. This study therefore aims to contribute to this knowledge gap by exploring the factors that shaped the acceptability and adoption of CHAs into the health system at district level during the initial phase of the integration process. This is part of a larger study examining the integration of CHAs into the health system in Zambia (see, for example, Zulu et al. [15]).

### The health system in Zambia

Like most health systems, the health system in Zambia operates at three levels: macro (national), meso (district and organizational) and micro levels (individuals/health post) [21]. This paper adopts the definition of health system by van Olmen et al. [22], which conceives the health system as consisting of governance and leadership, resources, service delivery, population, outcomes, and goals components.

There are six levels of care in the public sector and corresponding facilities in Zambia. The first four levels (namely outreach services, health posts, health centres, and level-1 district hospital) are located at the district level [23,24]. The other two levels are the provincial office and Ministry of Health national office. The health post is the lowest level health facility, and is often managed by a nurse. Due to limited human resources for health, support staff such as cashiers, cleaners and guards also help out with basic clinical tasks [24,25].

The Ministry of Health headquarters coordinates all health services in the country through the provincial and district offices. In the mid-1990s, Zambia implemented health sector decentralization which delegated powers to the District Health Management Team to supervise health services and human resources for health, and to coordinate decision making processes and the health information management system at the district level. In performing these tasks, the District Health Management Team works closely with health facilities and community structures such as the neighbourhood health committees. The neighbourhood health committees are responsible for mobilising people in the community for health promotion activities as well as providing information to the District Health Management Team on community health priorities [23].

**Figure 1:**
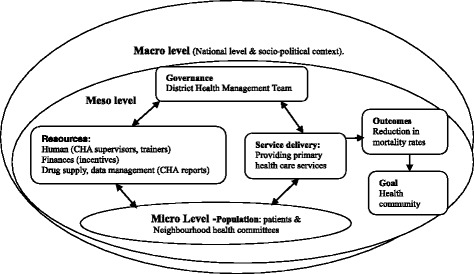
**Applied health system framework** **(adapted from van Olmen et al. [** 22 **]).**

As illustrated in Figure 1, which presents the elements of the health system [22] “elements of the system are highly interconnected with each other and what happens in one component often has ripple effects that affect other elements in multiple ways” [26]. For example, the availability of drugs at the health post will depend on existing health policies (leadership and governance) and finances (resources). Drug availability (resources) will affect CHAs’ (resources) ability to provide quality health care (service delivery) to the community (population). Service provision affects mortality rates (outcome) and, subsequently, the overall health status of the community (goal).

### Theoretical framework

The process of integration has been explained by different theories [11,27-29]. The diffusion of innovations theory, which concerns to how conditions increase or decrease the possibility that members of a social system will adopt an innovation [28], is one of the most widely used theories in health system and health services research [29-31].

According to this theory, diffusion is a process by which an innovation is communicated through certain channels over time among the members of a social system. An innovation is more likely to be accepted by the adopting system and thus would, be scalable if it has attributes of perceived relative advantage in relation to other options, compatibility with existing values and practices, and trialability, which is the degree to which an innovation can be experimented with on a limited basis. Other relevant attributes include the observability of the innovation, which is the degree to which the results can be visualized, and its perceived simplicity or ease of use [28].

The diffusion of innovations theory has been used in this study in order to facilitate understanding of the contextual/community processes and social factors that affected stakeholders’ acceptability and adoption of the community health assistants programme at district level in Zambia. The theory helped us explore how the attributes of the CHA programme interacted with the health system to either enhance or hinder its adoption at district level. In addition, the theory helped us draw lessons for the programme scale-up phase. Drawing from this theory, we developed the following assumptions: the innovation (CHA strategy) is more likely to be accepted in the district health system and would be scalable if it has attributes that are perceived to be relatively advantageous as compared with other community-based health workers; if it is compatible with values and principles of the health system; and if it is perceived as being simple to use by stakeholders in the health system. We also assumed that the degree to which the district is able to try out the CHA strategy and effectively observe the outcomes would influence the subsequent extent, rate and pattern of adoption [28,30].

## Methods

### The study site

The study was conducted in Kapiri Mposhi district, located in the Central Province of Zambia, about 185 kilometres north of the capital city, Lusaka. It has a population of about 240,000, with one hospital, four health centers and 22 health posts [23]. Kapiri Mposhi district was purposively chosen for this study because it is one of the rural districts where the CHA strategy has been piloted and it can easily be accessed from Lusaka. CHAs have been deployed in six health posts in the district. Some interviews were conducted with the CHA trainers in Ndola district on the Copperbelt province, the district where the CHA training school is located. Data collection was done by the authors.

### Data collection techniques

The interviews were conducted by the first author alone or together with the second and third authors, and analysed by all the authors; all of whom who have training and experience in qualitative research. The first author had experience of working with community-based health workers in Zambia and had conducted action research with the neighbourhood health communities as well as the District Heath Management Team in Kapiri Mposhi district in another research programme. Such prior experience enabled the researcher to easily create rapport and trust with the research participants thereby positively facilitating the data collection process.

#### Focus group discussions

Focus group discussions (FGDs) were conducted with members of the neighbourhood health committees who are representing the community. A total of six FGDs were conducted, one at each of the six health posts where CHAs have been deployed in the district. The health posts were identified from the records at the District Medical Office. Each focus group discussion had four participants who were the coordinators of the neighbourhood health committees in the health posts. The coordinators were included in the study because they were actively involved in recruiting CHAs as well as implementing the CHA programme at the community level. The rationale for the FGDs was to understand the community’s perspective of CHA services.

#### Key informant interviews

Key informant interviews were conducted with four trainers who were available at the training school during the study period, the CHA supervisor at each of the six health posts, and two staff in charge of implementing the CHA programme and other health services at the District Health Management Team level in Kapiri Mposhi district. Interviews with trainers were conducted in August 2012, while the other interviews were conducted from July to September 2013. Key informant interviews aimed at understanding CHA training, deployment, work and supervisory processes.

#### Review of documents

Documents reviewed included all reports and other materials on the piloting of the CHA programme, such as the CHA policy, newsletters, job descriptions, certificates, referral notes, CHA daily reports, and the implementation guide.

#### Data analysis

All interviews were recorded digitally and later transcribed verbatim by the first author. Analysis started while in the field, with familiarization of the data gained through reading and re-reading the material, and noting down initial ideas for analysis. We followed a thematic analysis approach, which “is a method for identifying, analysing and reporting patterns (themes) within data. It minimally organizes and describes a dataset in (rich) detail and goes further to interpret various aspects of the research topic” [32].

While familiarising ourselves with the data, a code manual was simultaneously inductively developed, based on the key questions and the theoretical underpinnings of the diffusion of innovations theory [28]. The code manual was then separately reviewed by all authors, by systematically comparing it to the dataset to arrive at the final code manual. The coding process, which involved matching the codes with segments of data selected as representative of the code, was carried out with NVIVO version 7 (QSR Australia). The coded data were then collated into potential themes. These were then reviewed through “checking if the themes work in relation to the coded extracts and the entire dataset”, before arriving at the final themes [32]. For instance, codes such as *training duration, curriculum,* and *certification* (under the ‘relative advantage’ diffusion of innovations condition) were first collated as *CHA competence* (potential theme), before being developed into *perceived good training.* Similarly, codes such *accessible services, drug availability* and *staff attitude* (under the ‘compatibility’ diffusion of innovations condition) were collated into the potential theme *quality health services*, which finally developed into *community healthcare expectations* (Table 1).

**Table 1:** **Selected themes describing relative advantage** (**A**) **and compatibility** (**B**) **DOI conditions**

**Code no**	**DOI condition**	**Name of code**	**Potential theme**	**Final theme**
A.1	Relative advantage	Training duration	CHA competence	Perceived good training
A.2		Curriculum		
A.3		Certification		
B.1	Compatibility	Accessible services	Quality health services	Community healthcare expectations
B.2		Drug availability		
B.3		Staff attitude		

Data from key informant interviews were then triangulated with other sources, such as the information gathered through FGDs, and the document review. It is important to note that this was an iterative analytical process which involved moving back and forth between data sources, codes and themes.

#### Ethical issues

Ethical clearance to conduct the study was obtained from the University of Zambia Biomedical Research Ethics Committee (IRB 0001131 of IORG 0000774, reference number 009-10-11). Verbal consent was sought from all study participants before conducting interviews or discussions. Further, detailed explanation of the research objectives was given to the participants, and they were informed that they were free to withdraw from the study at any point. Confidentiality during and after study was assured to the study participants. By withholding respondents’ personal details, it is not possible for readers to attribute views or statements to specific individuals.

## Results

This section presents the findings of the assessment of the early phase of the process of integrating CHAs into the health system at district level. The findings have been organised into five broad topics as per the diffusion of innovations model: relative advantage of CHAs, compatibility of the CHAs with health system components, trying out CHAs at district level, observing CHA pilot outcomes, and programme simplicity. The section starts by outlining the characteristics of the study participants.

### Socio-demographic characteristics

Twenty of the study participants were male while 16 were female. The respondents’ average age was 38 years, and their age ranged between 27 and 41 years. The duration of stay for CHA supervisors at the health posts varied between 6 months to 4 years while trainers had been at the training centre for about one and half years at the time of the study. All the members of neighbourhood health committees were appointed into the committees before the inception of the CHA programme in 2011. Two thirds of the twenty four neighbourhood health committee members who participated in the FGDs had previously worked as community-based health workers.

### Relative advantage of CHAs at district level

According to the diffusion of innovations condition on relative advantage, innovations that have features which are perceived as better than existing or previous similar programmes are likely to be more easily accepted and adopted. Perceived good training and broader responsibilities than the pre-existing community-based health workers were the main perceived advantages of CHAs.

#### Perceived CHA good training highly preferred by stakeholders

Most respondents indicated that they value health workers who have been through a training programme whose curriculum is developed by institutions registered with Ministry of Health. They stated that such a curriculum often has the right content to enable individuals to perform effectively. The awareness by stakeholders that the CHA curriculum, unlike that for other community-based health workers, was developed by major institutions responsible for training health workers in Zambia positively fostered acceptance and adoption of the CHAs at district level. These institutions included the General Nursing Council of Zambia, Health Professional Council of Zambia, University of Zambia, and Lusaka School of Nursing. Further, the awarding of training certificates by the Examination Council of Health Service to CHAs, which never applies to other similar cadres, made people view CHAs as more competent than the other cadres.

*“Acceptance of CHAs by the community is not a problem because their curriculum is good as it was developed by major training institutions in Zambia.”* (CHA trainer 2, female).

Staff and community members further reported that they prefer health workers who have been trained for not less than a year. They explained that training programmes that last for just a few months do not provide a broad knowledge base to trainees. They also stated that shorter training programmes do not provide trainees with sufficient time to practice what they learn.

*“The most important thing to remember is that unlike the training for the other community health workers, the CHA’s training is longer…. It runs for 1 year.”* (Neighbourhood health committee FGD 1, female participant 2).

#### CHA broader responsibilities attractive to stakeholders

The ability by CHAs to perform more tasks than the other community-based health workers due to their enhanced training was an additional advantage. It was reported that the other cadres only perform a few tasks, such as conducting awareness campaigns for HIV/AIDS, counselling, testing for malaria, and providing home-based care. However, CHAs conduct multiple tasks, including sensitisation campaigns on prevention of malaria, enhancing sanitation standards, use of family planning methods, and HIV/AIDS prevention. They also develop registers of community members, help deliver pregnant mothers, and test and treat such conditions as malaria, eye infections, diarrhoea, and respiratory tract infections. Trainers confirmed that CHAs are equipped with skills to undertake basic tasks usually conducted by nurses, clinical officers and environmental health technicians.

However, the limited involvement of CHAs in some health posts adversely affected the perceptions of CHAs among some community members. In some health posts, support staff such as cashiers and cleaners continued performing clinical tasks that CHAs could have been tasked with. The neighbourhood health committees complained that this limited involvement had made some people think that CHAs are less competent than support staff. At one health post it was reported that the neighbourhood health committee members called for a meeting to discuss the problem of inadequate involvement of CHAs.

*“We have two CHAs who were trained. But to our surprise, they are not allowed to give medicines. They just watch support staff give medicines.”* (Neighbourhood health committee FGD 2, male participant 1).

### Compatibility with district health system

Compatibility refers to how well the innovation is attuned to existing similar programmes, relevant bodies, and practices within the health system. It is also concerned with the extent to which the innovation is in line with community expectations.

#### CHA programme not a major shift

The analysis of data showed that the CHA concept was perceived as not being very different from existing community-based health worker approaches. This is because it shares some of the main features that characterise the community-based health workforce in the district. These include the involvement of community structures and leaders in selecting CHAs. Others are the requirement that one should be resident in the community in order to qualify as a CHA, and that the individuals should provide health services in the community where they reside.

#### CHA programme compatible with community healthcare expectations

The provision of basic services in the community was highly appreciated by community members. According to the neighbourhood health committee members, this approach was compatible with community expectations of what good health care services should include. It was reported that the community prefers services that are easily accessible. Delivering services in the community helps those who cannot easily travel to health facilities, such as the young, the disabled, and the elderly.

*“We are happy with the CHAs because they have brought health services close to our homes.”* (Neighbourhood health committee FGD 3, female participant 4).

However, data showed that most health posts had difficulties in adhering to the requirement that CHAs should spend about 80% of work time in the community, as this approach was not compatible with health post realities. Shortages of trained staff at health posts made it difficult for supervisors to allow CHAs to work in the community. Five of the six health posts had only one clinically trained member of staff (excluding CHAs), while the other health post had CHAs as the only trained staff. It was reported that some CHAs spend either 50% or more of their work time at the health post. Shortages of trained staff also made it difficult for supervisors to regularly monitor CHAs activities in the community.

*“Considering that staffing levels are poor, I decided that CHAs should do 80% work at the health post and 20% in the community.”* (CHA supervisor 1, male*).*

The inability of CHAs to always carry drugs to the community, due either to drug shortages at the health post or the refusal of some supervisors to allow them to carry drugs, limited the compatibility of the CHA concept with community health care expectations. The lack of drugs made it impossible for CHAs to treat common illnesses such as malaria in the community, and the neighbourhood health committee members feared that if not addressed, the situation may make many community members lose confidence in CHA services, as a few had already started complaining.

*“But the complaint in the community is that CHAs are unable to treat some illnesses like malaria as they do not have drug kits.”* (Neighbourhood health committee FGD 5, male participant 1).

#### CHA programme compatible with practices for professional health workers

Alignment of CHAs with existing professional bodies responsible for human resources for health in Zambia helped to facilitate the integration of CHAs into the health system. Review of records showed that CHAs are registered with and have practising certificates from the Health Professional Council of Zambia. Registration with professional bodies is important because it makes the new cadre adopt existing practices for professional health workers. Adoption of these practices by CHAs helped the other professional health workers not to consider CHAs as different from them. Some supervisors reported that the registration status made them confident to delegate tasks to CHAs.

*“I allow CHAs to perform some tasks because I know that are answerable to the Health Professional Council of Zambia.*” (CHA supervisor 6, male).

### Trying out CHAs at district level

Trialability refers to the extent to which an innovation can be tested and the lessons drawn from the testing process used to inform the scaling up or full implementation process. The CHA strategy is being tested through the pilot phase. The plan is to pilot one group of CHAs up to 2013, and recruit another group after evaluating the pilot phase.

#### Difficulties in trying out the CHA programme

Review of the CHA strategy and analysis of interviews with two District Health Management Team members showed that while a structure is in place at national level to facilitate the piloting process, no parallel structure has been put in place at the District Health Management Team level. The MoH formally appointed a strategic team at the national level to facilitate the process of formulating and piloting the strategy. The structure has four sub-committees, namely curriculum, logistics, monitoring and evaluation, and budgeting. Its role is to update management at the national level on the progress of the CHA strategy. However, there has been no equivalent formal structure at District level which limited integration of CHAs into the governance system of the District Health Management Team. The problem has been compounded by the fact that CHAs report to supervisors at the health post and also directly to the National Office, but not through the District Health Management Team as is the case with other professional health workers. Reports from CHA supervisors were also submitted directly from the health posts to the national level, but not through the District Health Management Team, and CHA supervisors were not part of the District Management Team. These issues affect trialability because they limit information flow about CHA activities and services between the District Health Management Team and CHAs. A lack of information at the District Health Management Team level makes it difficult for them to learn from CHA activities and respond to the challenges they face.

Another challenge was that there were some supervisors who were not familiar with the CHA programme. This is was due to the movement to other sites of supervisors who had training about the CHA programme. Inadequate knowledge of the CHA programme made it difficult for supervisors to manage their CHAs, while the lack of a comprehensive structure for coordinating CHAs made it difficult for the District Health Management Team to continuously train new supervisors about the CHA programme. Ineffective supervision affects trialability because it leads to limited documentation of CHA activities for decision-making purposes. An illustration of supervisors’ insufficient knowledge included uncertainties regarding the tasks or drugs that CHAs should be allowed to handle.

*“Sometimes CHAs come and ask for antibiotics to use. But am a little sceptical giving them drugs to administer because I don’t know the extent of their training.”* (CHA supervisor 2, male)*.*

### Observability of CHA activities at district level

Observability refers to indicators for ascertaining programme success or failure. Observable indicators for the CHA strategy include the contribution of CHAs towards increased antenatal visits, detections of respiratory tract infections, treatment of TB cases and malaria, use of bed nets and community sanitation standards, as well as reductions in infant and maternal mortality rates within the catchment areas. These are assessed through the CHAs’ own reports and monthly reports from their supervisors that are sent direct to the national level. CHAs provide referral notes to patients which can be used to track the patients that have been advised to visit facilities by CHAs.

#### Challenges in observing the CHA work

As in the case of CHA programme management, while a monitoring and evaluation committee is in place at national level to document programme outcomes, no monitoring and evaluation structure is in place at District Health Management Team level. The national level accesses information on programme indicators from the CHAs themselves and from their supervisors. Some informants feared that the limited involvement of existing district level monitoring and evaluation systems that document programme outcomes would result in challenges in observing the effect of CHAs’ work. Further, while routine monitoring was the responsibility of the national committee, it was reported that the committee had not yet visited the health posts from the time CHAs were deployed.

*“We are still waiting for monitors to come from the national level so that we can share with them some of challenges that we are experiencing in supervising CHAs.”* CHA supervisor 4, female).

### Simplicity of integrating CHAs at district level

Programme simplicity refers to how easy it is for stakeholders to understand the programme concept, to manage or control programme process, and to explain or interpret key programme issues.

#### Limited sense of programme ownership

Unlike other professional health workers, who only report to the MoH, CHAs are also accountable to community structures such as the Neighbourhood Health Committees, while at national level, they must report both to the Ministry of Health and to the Clinton Health Access Initiative, which has provided the strategy with technical support. This involvement of multiple stakeholders in handling CHA affairs meant that CHAs were perceived as a special group of workers who were not totally under the Ministry of Health. Interviews suggested that it was partly because of these uncertainties that CHAs in some health posts were not included in staff lists, and were not invited for meetings. Limited programme ownership affected programme simplicity as it made it difficult for the district to confidently ascertain the extent to which they could control the programme and of flexibility in modifying it.

*“We have been told that CHAs are under the Ministry of Health, but unlike other health workers, they are also controlled by the other groups. We are therefore not sure if they are totally under the Ministry of Health.”* (CHA supervisor 3, female).

#### Erratic and non-payment of incentives

The other challenge with regards to programme flexibility was about non- or erratic payment of CHA monthly allowances. Although CHAs have signed contracts which indicate that they are entitled to monthly incentives, supervisors informed that five CHAs had never received any payments over the previous nine months while the other seven had only been paid for about four of these months. This was a complex issue because no-one seemed to have a proper explanation for it, and as a result, the District Health Management Team and supervisors could also not effectively explain to CHAs about the cause of the problem and what could be done to resolve it.

## Discussion

This paper has reviewed how the early stage of the process of integrating community health assistants (CHAs) in the health system at district level in Zambia has evolved. The perceived good training of CHAs, and the broader responsibilities that they could consequently take, as compared to other community-based health workers, facilitated the integration process. These improvements made people perceive CHAs as being more competent and useful than existing, similar cadres. Further, the compatibility of the CHA concept with practices that underpin the existing community-based health workforce, with professional health workers, and with community expectations of quality health care, also positively influenced the integration process. Alignment of new innovations with prevailing practices in health system elements is important because it reduces possible conflicts which can either delay or distort the integration process. Such alignments appear to have provided CHAs with a clear advantage over the existing cadres, a situation which according to the diffusion of innovations theory, significantly influences the rate and pattern of integration [28,29,31,33].

The diffusion of innovations theory emphasises the testing of new innovations on a limited basis in order to observe or ascertain compatibility levels before scaling up innovations. This helps in identifying bottlenecks to integrating innovations at a larger scale [28] . Trialability has been conducted through piloting the CHA strategy at district level. Our data suggest, however, that limited integration of CHAs in the district health governance system affected the trialability process. There appears to be a disconnection in the level of integration into the governance health system component between national and district levels. While there is an elaborate structure to coordinate CHA activities at the national, macro level, nothing is in place at the district or meso level. This limited integration has constrained the successful flow of information on programme outcomes from CHAs to the District Health Management Team, which has limited the potential for learning lessons from the process, and reduced flexibility in programme implementation.

Further, the district faced difficulties in providing on-going training for CHA supervisors. Limited knowledge of the programme has therefore made it difficult for some supervisors to fully involve CHAs in duties and to document CHA activities at the heath posts, and this has limited programme observability. A recent assessment of the CHA programme in four districts in Zambia also showed that there has been limited involvement of the CHAs in some health posts which was partly attributed to not all CHAs being formally introduced to the health post staff, a situation which has left some supervisors unsure of the roles of CHAs [15]. This scenario effectively illustrates the interconnectedness of the different levels of the health system, insofar as what happens at the meso level has ripple effects that affect other elements on the micro level [26]. The findings support Atun *et al.* [11]’s view that the integration can happen differently at various levels in health system depending on the prevailing governance arrangements and support systems.

The findings of our study moreover demonstrate that limited integration of CHAs into the district health governance system as well as the involvement of multiple stakeholders in managing CHA activities has affected people’s notions of programme ownership. The involvement of a variety of different stakeholders has made it difficult for CHA supervisors to define managerial boundaries. In addition, requiring CHAs to directly submit reports to the national office appears to have created a communication system which is not compatible with the existing system at the district level. These shortcomings could affect programme sustainability as they also seem to be incompatible with existing decentralisation policy principles which have delegated powers to the District Health Management Team so that they can manage all health matters at district level [23]. Incompatibilities could further contribute towards insufficient documentation of programme evidence, thereby limiting an effective learning process during the scaling up phase.

Furthermore, failure by the national level to regularly pay CHA incentives and relay information to the district regarding reasons for delayed payment has made it difficult for the District Health Management Team and CHA supervisors to effectively respond to CHA concerns about their incentives. A recent evaluation of the CHA programme suggests that the delay has been due to lapses in administrative processes and limited communication from the Ministry of Health to the district level which resulted into leaving districts unsure of what to communicate to supervisors and CHAs [34]. This could affect service delivery if not addressed, as studies on similar programmes such as the Ethiopian Health Extension Workers [12,13] and Lady Health Worker program in Pakistan [7] suggest that inability to regularly pay remuneration emerges as an impediment to implementation of initiatives that utilise community-based health workers’ services [26]. To ensure sustained and effective service provision at community level, various elements of the health system, including finances, must be strengthened [35,36].

In addition to demonstrating that the limited integration of CHAs into the district governance system has affected processes at the health post level, the study has also shown that bottlenecks at a particular health system level can have ripple effects across elements at the same level. For example, insufficient drugs at the health post affected CHAs’ ability to effectively deliver services in the community, a finding consistent with a recent process evaluation of the CHA pilot phase in four other districts [34]. This situation may distort the integration process over time, as the literature suggests that lack of drugs may make community members to lose trust and respect in the services provided by community-based health workers [37]. For instance, Afsar and Younus [38] [p. 1] reported that “poor supply caused embarrassment and made lady health workers suspect in the eyes of the community because they were accused of selling drugs and contraceptives in the market”. We therefore support Lehmann and Sanders’ [39] [p. vi] argument that although community-based health workers are a good investment, they are “neither the panacea for weak health systems nor a cheap option to provide access to health care for underserved populations”.

Our study further shows that limited involvement of CHAs at the health post created doubts among some community members about their competence levels. It was feared that if not addressed, these doubts could be increasingly shared by many people, and could possibly undermine community perceptions about the relative advantage of CHAs. These fears could be explained by the social narrative theory which states that when an innovation is launched, people develop impressions about it and share these with others. As the narrative goes on, people start taking up different positions in relation to the innovation, based on what they hear, and this often shapes the pattern of integration [40].

Perhaps the difficulties in integrating human resources for health innovations into the health system in the initial stages could be normal outcomes of the integration processes. Creation of a new cadre has the potential to pose challenges for acceptance, coordination and sustainability, because the adopters may be uncertain about the benefits and risks of the innovation [41,42].

Identifying and reducing the challenges to the integration process would require adopting a systems thinking approach. This demands careful consideration of possible consequences of an innovation through team work and collaborative thinking [1]. It entails critically considering in an iterative and systematic way the interactions between health system elements at macro, meso and micro levels [21], in order to simulate desirable health system behaviours under explicit assumptions and conditions [43]. It also involves “identifying the possible intended and unintended implications” [26] [p. 9] of the innovation on health system actors and institutions. The approach could effectively help in identifying the possible consequences of limited integration of CHAs into the district health governance system on the lower health system levels, while also helping to develop measures that positively facilitate the acceptability and adoption of processes at various levels of the health system.

### Trustworthiness

Trustworthiness of the study was enhanced through attending to aspects of credibility, dependability and transferability of our findings [44,45]. The credibility and dependability of findings were strengthened through systematically and comprehensively reviewing the data and inductively coding and categorization [46]. We also aimed to enhance credibility and dependability of findings by separately sharing the codes and categories among the researchers, and individually reviewing them and finally discussing the individual insights of the data to develop the themes. The complementary backgrounds and qualifications of the researchers (anthropology and public health) helped in improving trustworthiness of data and its analysis and interpretation. We aimed to strengthen transferability by providing a rich description of the phenomena, informants, the procedures of data collection and analysis, and by providing quotations in the text representing a variety of informants [47,48].

Conducting only one FGD per health post with the neighbour hood health committees, with the majority having previously worked as community-based health workers, and not including the general community members, denied the study some important perspectives on the CHA integration process; such as the relationship between CHAs and community members who do not play any roles in the supervision or recruitment of CHAs. However, by systematically highlighting context-specific processes of integrating CHAs in the health system, this work may provide a basis for analytic generalizations that could provide useful insights not only to the Ministry of Health in Zambia but also to other low and middle income countries. As a follow up, we recommend conducting a mixed methods study in Zambia with a larger sample comprising different study populations (also including caregivers of children treated, CHAs, etc.) as well as comprehensive checking of referral notes/records of CHAs or health facility records on referrals received in order to ascertain the impact of the CHA programme on health outcomes.

## Conclusion

This study has sought to provide an assessment of the acceptability and adoption of community health assistants (CHAs) into the Zambian health system at district level during the early pilot phase of the strategy. The study was guided by the diffusion of innovations theory. Our results suggest that there were differences in the level and pattern of integration between the national and district health governance system. The perceived relative advantage of CHAs with other types of community-based health workers as well as their compatibility with professional health staff and community health care expectations positively influenced the integration process.

However, limited integration of CHAs in the district health governance system affected programme simplicity, trialability and observability. For example, the limited integration constrained effective documentation of programme outcomes, supervisory processes, and reduced a sense of district level programme ownership. Further, neither the district nor the CHA supervisors could effectively explain to the CHAs reasons for delayed and non-payment of CHA incentives. These bottlenecks affected effective health service delivery both at the community and health post level.

Successfully integrating CHAs into the health system would require adopting a systems thinking approach, as health systems are interconnected, dynamic and complex in nature. This approach could help identify ripple effects that result from the limited integration of CHAs into the district health governance system. It would also help to develop measures that can facilitate simultaneous changes at different health system levels with a view towards effectively supporting the integration process.

## Abbreviations

CHAs: Community health assistants
FGDs: Focus group discussions
